# Investigation of the Effects of Preoperative Hydration on the Postoperative Nausea and Vomiting

**DOI:** 10.1155/2014/302747

**Published:** 2014-01-20

**Authors:** M. Selçuk Yavuz, Dilek Kazancı, Sema Turan, Bahar Aydınlı, Gökçe Selçuk, Ayşegül Özgök, Ahmet Coşar

**Affiliations:** ^1^Duatepe Polatlı State Hospital, Ankara, Turkey; ^2^Türkiye Yüksek İhtisas Education and Research Hospital, Anesthesiology and Reanimation Clinic, Kızılay Street No. 4, Samanpazarı Altındağ, Ankara, Turkey; ^3^Gülhane Military Medicine Faculty, Anesthesiology and Reanimation Department, Ankara, Turkey

## Abstract

*Introduction*. Postoperative nausea and vomiting (PONV) after laparoscopic cholecystectomy operations still continue to be a serious problem. Intravenous fluid administration has been shown to reduce PONV. Some patients have higher risk for PONV described by APFEL score. In this study, our aim was to determine the effects of preoperative intravenous hydration on postoperative nausea and vomiting in high Apfel scored patients undergoing laparoscopic cholecystectomy surgery. *Patients and Methods*. This study is performed with 50 female patients who had APFEL score 3-4 after ethics committee approval and informed consent was taken from patients. The patients were divided into 2 groups: group 1 (P_1_): propofol + preoperative hydration and group 2 (P_2_): propofol + no preoperative hydration. *Results*. When the total nausea VAS scores of groups P_1_ and P_2_ to which hydration was given or not given were compared, a statistically significant difference was detected at 8th and 12th hours (*P* = 0.001 and *P* = 0.041). It was observed that in group P_1_, which was given hydration, the nausea VAS score was lower. When the total number of patients who had nausea and vomiting in P_1_ and P_2_, more patients suffered nausea in P_2_ group. *Discussion*. Preoperative hydration may be effective in high Apfel scored patients to prevent postoperative nausea.

## 1. Introduction

Postoperative nausea and vomiting (PONV) after laparoscopic cholecystectomy operations still continue to be a serious problem. In the literature, the incidence of PONV is reported to be around 10%. However, with the addition of any one of the risk factors, the ratio may rise to proportions that range between 21% and 79%. Intravenous fluid administration has been shown to reduce PONV. Some patients have higher risk for PONV described by APFEL score. The simplified Apfel score includes four factors: female gender, no smoking, postoperative use of opioids, and previous PONV or motion-sickness in patients' history that each one of these risk factors were scored with 1 point and supposed to elevate the PONV-incidence about 20%.

In order to prevent PONV, patient-controlled antiemesis, oxygen administration, elimination of pain, and use of antiemetic agents, such as sedatives, anxiolytics, antimuscarinics, corticosteroids, antagonists of dopamine D2, serotonin antagonists, and provision of adequate hydration, can be applied. In the literature, there is different information about the effectiveness of application of intravenous hydration on early and late nausea and vomiting.

In this study, our aim was to determine the effects of preoperative intravenous hydration on postoperative nausea and vomiting in patients undergoing laparoscopic cholecystectomy surgery.

## 2. Patients and Method

Following the approval of local ethics committee, 50 women who will undergo elective laparoscopic cholecystectomy with ASA physical statuses I-II were included in the study. This study is performed with 50 female patients who had APFEL score 3-4, after ethics committee approval and informed consent was taken from patients. However, patients who have body weight > 100 kg, who are older than 60 years old or younger than 18 years of age, smokers, pregnant or lactating, as well as patients who have cardiovascular, neurological, pulmonary, or endocrine diseases, patients who are unable to tolerate the process of the operation, patients who have extreme anxiety immediately after the operation, patients who have intraoperative excessive bleeding, patients who are passed on to open surgery and patients who use antiemetic drugs within 24 hours were all excluded from the study.

The patients were divided into 2 groups.

 Group 1 (P_1_): propofol + preoperative hydration:
 preoperative hydration: preoperative 1 hour, hydration with 15 mL/kg Ringer's lactate;
 induction: propofol 2-3 mL/kg + 1-2 g/kg fentanyl + 0.6–0.8 mg/kg rocuronium;
 intraoperative: 5 mL/kg/h Ringer's lactate;
 Group 2 (P_2_): propofol + no preoperative hydration:
 preoperative: preoperative 1 hour, basal fluid requirements were met with 2 mL/kg Ringer's lactate;
 induction: propofol 2-3 mL/kg + 1-2 g/kg fentanyl + 0.6–0.8 mg/kg rocuronium;
 intraoperative: 5 mL/kg/h Ringer's lactate.

Patients were received into the operation room without premedication. ECG, oxygen saturation (SpO_2_), noninvasive blood pressure, and end tidal carbon dioxide (ETCO_2_) monitoring were performed. Ringer's lactate infusion was continued in all patients with 5 mL/kg/h. Following adequate preoxygenation, induction of anesthesia was performed in the proper way. In maintenance of anesthesia, 50% air + 50% oxygen + 2–2.5 MAC sevoflurane were used. Patients' heart rates, mean blood pressures, ETCO_2_ (keeping the range between 30 and 35 mmHg) and SpO_2_ values were recorded 15 min before and after induction. After removing the gallbladder, while hemostasis was achieved analgesia was achieved with im 75 mg diclofenac sodium, in all groups. After extubation, the hemodynamic followup of the patients who were taken to the recovery unit was performed by a physician who did not know the patient groups. The patients' nausea, vomiting, and pain were examined by the same physician at 0, 0.5, 1, 4, 8, 12, and 24th hours. As in a routine practice, in patients with VAS value of 5 or more, 10 mg metoclopramide was administered slowly from intravenous line. At the same intervals, their pain was assessed with VDS. In case of VDS > 3, during the first 1 hour, 1 mg/kg pethidine HCL was injected intramuscularly. In patients who had been taken to their rooms, an additional dose of diclofenac 75 mg was administered IM to those with VDS > 3.

## 3. Findings

There was no statistical difference between the demographic characteristics of the patients. The statistical difference found between the total amounts of fluid given is due to the preoperative hydration administered to group 1 and group 2. Groups did not differ when compared among themselves (Table 1). Patients ASA values were found to be similar to the previous stories of carsickness and PONV. Moreover, there was no statistically significant difference between patients' systolic and diastolic blood pressures during preoperative, intraoperative, and postoperative periods.

In comparison of groups P_1_ and P_2_, there was a decrease in nausea VAS scores in the group that was given hydration in the early period (0-1 hours) though it was not statistically significant. In the late period (1–24 hours), at the 8th hour and 12th hours, there was a significant difference in nausea in terms of VAS values (*P* = 0.001, *P* = 0.041). Even if there was no significant difference in the subsequent hours, in the propofol group to which hydration was given, VAS values were found to be even lower (Table 2).

When the total nausea VAS scores of the groups to which hydration was given or not given were compared, a statistically significant difference was detected at 8th and 12th hours (*P* = 0.001 and *P* = 0.041). It was observed that in group P_1_, which was given hydration, the nausea VAS score was lower (Table 2).

When the total nausea and vomiting numbers of the groups which were given or not given hydration were compared, within the range of 1–24 hours, more nausea and vomiting were observed in the group to which hydration was not given (Table 3).

There was no significant difference between groups about the metoclopramide use (Table 4).

There was no significant difference between the groups in terms of postoperative pain.

## 4. Discussion

In this study, the effects of preoperative i.v. hydration on the incidence of postoperative nausea and vomiting are examined. Nausea and vomiting have a complex etiology which is affected by many factors such as age, gender, obesity, previous history of PONV, carsickness history, smoking, use of opioids during and after surgery, anesthesia and surgical techniques, and postoperative pain [1–5]. Apfel and coauthors stated that the most reliable independent predictors of PONV were female gender, history of PONV or motion sickness, nonsmoking, younger age, duration of anaesthesia with volatile anaesthetics, and postoperative opioids in their review [5]. As it was affected by so many variables, we tried to ensure maximum standardization in our study. In this way, demographic characteristics and the characteristics related to the operation were found to be similar in the groups. One of the important factors which is observed after general anesthesia and which affects the PONV is the surgical procedure. Since it is applicable in daily life and provides other benefits laparoscopic surgery is used routinely. Following laparoscopic surgery, PONV can be seen very frequently [5–7]. After intraperitoneal CO_2_ insufflation, increasing gradually abdominal pressure can cause regurgitation. Furthermore, a nasogastric tube which is inserted during laparoscopic surgery can also cause irritation and thus increase the risk of PONV. Having been absorbed, the insufflated CO_2_ joins the systemic circulation and may cause hypercarbia. The endogenous catecholamines which increase as a result of hypercarbia may also increase the risk of nausea and vomiting [8–11].

Several different drugs and techniques have been used in the prevention and treatment of postoperative nausea and vomiting. In our study, although no significant difference was observed in the incidence of nausea in early stages (0-1 hr), in the late period (1–24 hr) a significant difference was observed at the 8th and 12th hours in the groups which were given preoperative hydration. It was observed that hydration had to decreased mild nausea (nausea VAS 2–4) by 27%, moderate and higher nausea (nausea VAS > 5) by 36%. As for vomiting rates in the groups, a significant difference observed only in the first postoperative exit but not observed at other times, and the incidence of vomiting in the hydrated group was 8%, while in the nonhydrated group it was 18%. When the number of metoclopramide which was administered to two groups was considered, a significant difference in the amount of metoclopramide that was needed was observed only during the first 1 hour either following vomiting accompanied by severe nausea (nausea VAS > 10) or vomiting with no nausea at all. Subsequently, this significant difference disappeared and 12% of the hydrated groups and 46% of the nonhydrated groups needed metoclopropamide. When pain was analyzed in each of the four groups, no significant difference was observed in terms of VAS values, and finding moderate to severe pain with VAS ≥ 3 to be 12% in the hydrated groups and (20–24%) in the nonhydrated group as well as observing a reduction in pain close to 50% was evaluated as an important clinical outcome.

In the literature, Fiddian-Green, who applied the liquids prior to the induction of anesthesia, reported that in the early postoperative period there was no significant difference in the incidence of nausea and in late period there was a decrease in the postoperative nausea [12]. In the study carried out by Ali et al., it was stated that while the additional Hartmann solution given to a group at 15 mL/kg rate did not affect the early PONV very much as compared to the group given 2 mL/kg, it did prove effective in the late period [13]. On the other hand, in the study done by Adanır et al., it was reported that the groups which were given preoperative hydration (12 mL/kg HES or Ringer's lactate) had lower VAS scores at the postoperative 4th hour as compared to the control group (2 mL/kg); namely, through early hydration it was possible to reduce the incidence of PONV [14]. In our study, as in the previous studies, we also observed a significant decrease in the incidence of PONV and drop in VAS scores in the 24 hours following anesthesia.

Pain that develops postoperatively is one of the factors that increases the risk of PONV. In our study, no statistically significant difference was found in terms of postoperative analgesic use and postoperative pain. However, when the moderate to severe pain (pain VAS ≥ 3) values were considered in all study groups, even though the pain level of 12% in the hydrated group and 20–24% in the nonhydrated group may not to be considered as a statistically significant difference, it was evaluated as an important clinical outcome. While the study carried out by Chaudhary et al. [15] reported that preoperative hydration caused a slight decrease in postoperative pain scores and use of opioid, the study conducted by Spencer [16] did not identify any such relationship between hydration and postoperative pain.

That the additional fluid therapy reduces nausea mechanism still remains unknown. Perioperative hypoperfusion of intestinal mucosa and the subsequent development of possible ischemia may be one of the causes of PONV [17]. Hypovolemia that develops in the patients who are hungry before the induction of anesthesia does not improve so easily until postoperative period.

The additional fluid overload before the induction of anesthesia most likely reduces the volume deficit and brings the patients closer to normovolemia. In our study, we also found that intravenous hydration given preoperatively could reduce the incidence of PONV in relation to differences in gender, type of surgery and technique in anesthesia. Thus, we think that nausea and vomiting and the associated complications can be decreased, and moreover it will be possible to avoid the side effects that the use of antiemetics brings about in the process of treatment.

## Figures and Tables

**Table 1:** Demographic values of the patients.

	Group-I (P_1_) (*n* = 25)	Group-II (P_2_) (*n* = 25)	*P*
Age (year)	(43 ± 11.9)	(45 ± 10.2)	0.936
	[45 ± 20]	[47 ± 20]	
	Max: 60 Min: 19	Max: 60 Min: 31	

Weight (kg)	(71.28 ± 10.54)	(72.16 ± 11.73)	0.907
	[69 ± 11.5]	[72 ± 21.5]	
	Max: 93 Min: 55	Max: 95 Min: 55	

BMI (Kg/m^2^)	(27.55 ± 4.03)	(27.80 ± 3.83)	0.971
	[26.5 ± 3.45]	[27.5 ± 6.55]	
	Max: 36.30, Min: 20.70	Max: 34.9 Min: 20	

Total hydration (mL)	(1418 ± 244.4)	(474 ± 103)	0.000*
	[1400 ± 200]	[500 ± 150]	
	Max: 2000 Min: 950	Max: 700 Min: 200	

Surgery duration (min)	(39.12 ± 11.19)	(34.44 ± 9.12)	0.049
	[38 ± 11.5]	[32 ± 17.5]	
	Max: 61 Min: 23	Max: 50 Min: 21	

Anaesthesia duration (min)	(53.8 ± 10.67)	(50.20 ± 10.07)	0.058
	[49 ± 20]	[47 ± 20.5]	
	Max: 70 Min: 39	Max: 70 Min: 38	

*P < 0.05 statistically significant.

**Table 2:** Postoperative nausea VAS scores.

Zaman (saat)	Group-I (P_1_) (n = 25)	Group-II (P_2_) (n = 25)	*P*
0	(0.32 ± 0.47)	(0.72 ± 1.64)	0.849
	[0-1]	[0–6]	
0.5	(0.52 ± 0.58)	(0.56 ± 0.87)	0.707
	[0–2]	[0–3]	
1	(0.8 ± 0.81)	(0.64 ± 0.7)	0.414
	[0–4]	[0–3]	
4	(1.2 ± 2.29)	(2.12 ± 3.1)	0.323
	[0–10]	[0–10]	
8	(0.12 ± 0.33)	(0.96 ± 1.51)	0.001*
	[0-1]	[0–7]	
12	(0.12 ± 0.33)	(0.32 ± 0.47)	0.041*
	[0-1]	[0-1]	
24	(0.08 ± 0.27)	(0.28 ± 0.45)	0.068
	[0-1]	[0-1]	

*P < 0.05 statistically significant.

**Table 3:** Dispersion of vomiting according to groups.

Post op period (hour)	Group-I (P_1_) (*n* = 25)	Group-II (P_2_) (*n* = 25)	*P*
0	—	—	
0.5	—	—	
1	—	1 (4%)	0.387
4	1 (4%)	2 (8%)	0.780
8	—	—	—
12	—	—	—
24	—	—	—

Total	1 (4%)	3 (12%)	4 patient

**Table 4:** Metoclopramide use in groups 1 and 2.

Post op period (hour)	Group-I (P_1_) (*n* = 25)	Group-II (P_2_) ( *n* = 25)	*P*
0	—	2 (8%)	0.244
0.5	—	—	0.564
1	—	—	0.054
4	2 (8%)	5 (20%)	0.548
8	—	1 (4%)	0.286
12	—	—	0.106
24	—	—	—
